# PIMA™ point-of-care testing for CD4 counts in predicting antiretroviral initiation in HIV-infected individuals in KwaZulu-Natal, Durban, South Africa

**DOI:** 10.4102/sajhivmed.v17i1.444

**Published:** 2016-06-24

**Authors:** Mandisa Skhosana, Shabashini Reddy, Tarylee Reddy, Siphelele Ntoyanto, Elizabeth Spooner, Gita Ramjee, Noluthando Ngomane, Anna Coutsoudis, Photini Kiepiela

**Affiliations:** 1 Department of Paediatrics and Child Health, University of KwaZulu-Natal, South Africa; 2 Medical Research Council of South Africa, HIV Prevention Research Unit, South Africa; 3 Medical Research Council of South Africa, Biostatistics Unit, South Africa; 4 eThekwini Health Unit, eThekwini Municipality, South Africa

## Abstract

**Introduction:**

Limited information is available on the usefulness of the PIMA™ analyser in predicting antiretroviral treatment eligibility and outcome in a primary healthcare clinic setting in disadvantaged communities in KwaZulu-Natal, South Africa.

**Materials and methods:**

The study was conducted under the eThekwini Health Unit, Durban, KwaZulu-Natal. Comparison of the enumeration of CD4+ T-cells in 268 patients using the PIMA™ analyser and the predicate National Health Laboratory Services (NHLS) was undertaken during January to July 2013. Bland-Altman analysis to calculate bias and limits of agreement, precision and levels of clinical misclassification at various CD4+ T-cell count thresholds was performed.

**Results:**

There was high precision of the PIMA™ control bead cartridges with low and normal CD4+ T-cell counts using three different PIMA™ analysers (%CV < 5). Under World Health Organization (WHO) guidelines (≤ 500 cells/mm^3^), the sensitivity of the PIMA™ analyser was 94%, specificity 78% and positive predictive value (PPV) 95%. There were 24 (9%) misclassifications, of which 13 were false-negative in whom the mean bias was 149 CD4+ T-cells/mm^3^. Most (87%) patients returned for their CD4 test result but only 67% (110/164) of those eligible (≤ 350 cells/mm^3^) were initiated on antiretroviral therapy (ART) with a time to treatment of 49 days (interquartile range [IQR], 42–64 days).

**Conclusion:**

There was adequate agreement between PIMA™ analyser and predicate NHLS CD4+ T-cell count enumeration (≤ 500 cells/mm^3^) in adult HIV-positive individuals. The high PPV, sensitivity and acceptable specificity of the PIMA™ analyser technology lend it as a reliable tool in predicting eligibility and rapid linkage to care in ART programmes.

## Introduction

Poor rates of linkage to care for those with low CD4+ T-cell counts, eligible for antiretroviral therapy (ART), have been reported in several African cohort studies.^1,2,3,4,5^

Several attrition steps exist in the continuum of care pathway: patients lost to care between testing HIV-positive and going for a CD4 test^6^; CD4 test result not available and/or lost^7,8^; patient not returning for their CD4 test result; and lack of uptake of care from eligibility to initiation of ART even in those who return for test results.^9^ These challenges may be overcome by point-of-care (POC) testing^10^, resulting in less attrition over time.^2,4^ It has been suggested that POC CD4 testing in those who do not return for their results would potentially increase enrolment pre-ART.^9^ POC CD4 testing was shown to modestly increase linkage to care and reduce pre-treatment loss to follow-up in fixed and mobile clinics.^2,11,12,13^ Factors contributing to pre-treatment loss to follow-up have been previously documented.^8,14,15,16,17^

The Alere PIMA™ POC has been evaluated against the ‘gold standard’ flow cytometry platforms, for example, Beckman Coulter using panleucogating (PLG)^18^; BD FACS count^19,20,21^; PARTEC Cytoflow™^19,20^; Guava and BD FACS Calibur^20,21,22,23,24^ for the enumeration of CD4+ T-cells in HIV-1-infected adults and in HIV-1-infected pregnant women.^22^

This study assessed the accuracy, sensitivity and specificity of the Alere PIMA™ POC analyser in CD4+ T-cell count enumeration compared to the predicate South African National Health Laboratory Services (NHLS) flow cytometry test (Beckman Coulter) and its potential operational role as a predictor of ART eligibility in a primary healthcare clinic (PHC) in Durban, South Africa.

## Materials and methods

The study occurred at Lancers Road PHC, a facility under the eThekwini Health Unit, situated in the centre of the convergence of the taxi rank in the city centre of Durban. This PHC offers HIV Counselling and Testing (HCT) – approximately 900 per month to walk-in patients who receive pre- and post-test counselling and CD4 testing for the staging of HIV-1-infected disease to determine eligibility for ART. Patients are advised to return after 7 days for their CD4 results. As per the SA HIV and AIDS guidelines^25^ operating at the time of this study, patients with a CD4+ T-cell count ≤ 350 cells/mm^3^, upon their return, were medically assessed, and education and counselling undertaken prior to ART initiation. Those ineligible for ART, viz CD4+ count > 350 cells/mm^3^, were counselled to return 6 monthly for CD4+ T-cell count testing and for further medical assessment. Eligible patients, who did not return for results, were contacted telephonically to ascertain whether they had been initiated on ART elsewhere, and if not, they were encouraged to return for further care.

### Testing of venous blood samples

Routine CD4+ T-cell enumeration is conducted at the NHLS one day after the blood draw via Beckman Coulter flow cytometry using PLG methodology, the standard of care in this setting as described previously.^26^ During January 2013 to July 2013, in 268 patients, an extra 2 mL of venous blood was drawn from the same blood draw as the routine NHLS test into another ethylenediaminetetraacetic acid (EDTA) tube for the comparison of the enumeration of CD4+ T-cells using the Alere PIMA™ technology (Alere Health Care, Waltham, Massachusetts). PIMA™ POC CD4+ T-cell enumeration was conducted by a laboratory technician who pipette-filled the PIMA™ cartridges. Three PIMA™ analysers were used in this study. CD4+ T-cell count enumeration was performed in a subset of 100 samples using the FACS Calibur.

### Quality control and/or precision of PIMA™ analysers

Quality control and routine PIMA™ analyser maintenance were performed daily as per manufacturer’s guidelines: one control has low CD4+ T-cell counts (115 cells/mm^3^ – 235 cells/mm^3^) and the other has normal CD4+ T-cell counts (719 cells/mm^3^ – 1355 cells/mm^3^). Daily quality control was conducted on all 3 analysers for the first 10 measurements when a new cartridge was used and over a period of 165 days (23 January – 25 March 2014). Accuracy and precision of the NHLS PLG testing was established in the NHLS laboratories by daily monitoring of instrument stability (Flow check TM, Beckman Coulter Miami, FL) and system performance verification using normal (394 cells/mm^3^ – 754 cells/mm^3^) and low (62 cells/mm^3^ – 206 cells/mm^3^) Immunotrol™ controls (Beckman Coulter, Miami, FL). The Addington NHLS laboratory participates in the NHLS proficiency testing panels and is accredited by the South African National Accreditation System.^27^

### Reproducibility of CD4+ T-cell enumeration across flow cytometry instruments

Comparisons of CD4+ T-cell enumeration was undertaken between flow cytometry instruments (PIMA™ POC analysers and the predicate NHLS) on 268 blood samples. Due to transport logistics, the NHLS laboratory performs testing the day after the blood draw. Therefore, a subset of 100 blood samples were tested by the PIMA™ analyser, FACS Calibur and the NHLS to ensure that differences observed between the PIMA™ analyser versus NHLS were not due to CD4 testing performed on the next day in the NHLS laboratory. CD4+ T-cell enumeration using the FACS Calibur reference method^28^ was undertaken on the same blood sample tube as the PIMA™ POC analyser at the Medical Research Council Central laboratory, which participates in the United Kingdom National External Quality Assessment Scheme (UK NEQAS) quality assessment programme.

### Predictions of benefit of PIMA™ POC CD4 test results for ART eligibility and linkage to care

Prediction of the benefits of the PIMA™ POC CD4 testing in terms of ART eligibility and decision making was undertaken. Additionally, an assessment was undertaken to determine whether HIV-infected individuals return for their CD4+ test result and how many are lost to follow-up between ART eligibility and initiation.

The protocol was approved by the Biomedical Research Ethics Committee, University of KwaZulu-Natal (BE 212/11) and the eThekwini Research Ethics committee (28 November 2011). Written informed consent was obtained from patients > 18 years of age enrolled in the study.

#### Statistical analysis

It was determined that a sample size of 254 HIV-positive patients would be required to detect a difference of 15 cells/mm^3^ between the results of the PIMA™ POC analyser and the conventional test with 95% probability and 80% power assuming the standard deviation of difference in means is 85. In order to allow for potential problems with samples, the sample size was increased by 14 patients giving a sample size of 268.

#### Statistical methods

Pairwise comparison of the PIMA™ analysers was conducted using *t*-tests. To assess the precision of the control cartridge within each of the three PIMA™ analysers, the %CV was calculated for the 10 observations (intra-day reproducibility) and over a period of 165 days (inter-day reproducibility) at low and normal beads.

The percentage similarity (% SIM) model, Bland-Altman (BA) plots, limits of agreement (LOA) and Lin’s concordance correlation coefficient were used to assess agreement between PIMA™ analysers, FACS Calibur and NHLS.^29^

To assess the diagnostic accuracy of CD4+ T-cell counts by the PIMA™ POC analysers in identifying ART eligibility, sensitivity, specificity, false-negative (FN) and false-positive (FP) rates, positive predictive value (PPV) and negative predictive value (NPV) were computed for the ART initiation thresholds of ≤ 200 cells/mm^3^, ≤ 350 cells/mm^3^ and ≤ 500 cells/mm^3^ CD4+ T-cells. All analyses were performed using STATA (Statacorp, College Station, TX, USA) statistical version 13.

## Results

### Reproducibility of results of PIMA™ machines used in this study

There was high reproducibility and instrument precision (%CVs < 5%) within PIMA™ analysers 1, 2, 3 of the control cartridges over a replicate set of 10 bead analyses and over time (*n* = 165 days; 23 January – 25 March 2014). The bead quality control (QC) count for low and normal bead cartridges showed median %CV results for the 10 same-day observations of 2.13%, 1.28%, and 1.41% and 0.86%, 1.36%, and 0.96% for analysers 1, 2, and 3, respectively. Bead QC counts for low and normal bead cartridges showed median %CV results over the 165 days of 1.75%, 1.70%, and 1.86% and 1.14%, 1.67%, and 1.30% for PIMA™ analysers 1, 2, and 3, respectively.

System performance verification using normal (394 cells/ mm^3^ – 754 cells/mm^3^) and low (62 cells/mm^3^ – 206 cells/mm^3^) Immunotrol controls for the NHLS PLG testing was < 6%.

The majority (218/268) of HIV-1-positive individuals undergoing CD4+ T-cell count testing were women of whom 25% were 25–29 years, whereas the majority of the men were older than 30 years (Table 1). There was no significant difference in the median CD4+ T-cell count between men and women performed by the NHLS versus the PIMA™ POC analyser, although the median CD4+ T-cell count was higher in the latter. According to the NHLS versus PIMA™ POC, 81% versus 80% of HIV-positive individuals were eligible for ART initiation (≤ 500 cells/mm^3^), of whom 82% versus 84% were males and 81% versus 79% were females, respectively.

**TABLE 1:** Characteristics of HIV-1-positive individuals undergoing CD4+ T-cell count enumeration.

Patient characteristics	Female†			Male‡			Total§		
		
	*n*	%	Range	*n*	%	Range	*n*	%	Range
Median age (IQR), years	32	-	26–37	33	-	30–40	32	-	27–38
18–24	39	17.90	-	4	8	-	43	16.00	-
25–29	54	24.77	-	8	16	-	62	23.10	-
30–34	46	21.10	-	18	36	-	64	23.90	-
35–39	44	20.20	-	7	14	-	51	19.00	-
> 40	35	16.10	-	13	26	-	48	17.90	-
Median (IQR) NHLS CD4 count cells/mm^3^	292	-	184–453	254	-	151–387	286	-	176.5–444.5
Number (%) NHLS ≤ 350 cells/mm^3^	130	60	-	34	68	-	164	61.19	-
Number (%) NHLS ≤ 500 cells/mm^3^	176	81	-	42	82	-	218	81	-
Median (IQR) PIMA™ CD4 count cells/mm^3^	328	-	204–451	308	-	179–419	322	-	204–449
Number (%) PIMA™ ≤ 350 cells/mm^3^	114	52.30	-	31	62	-	145	54.10	-
Number (%) PIMA™ ≤ 500 cells/mm^3^	173	79	-	42	84	-	215	80	-

IQR, interquartile range; NHLS, National Health Laboratory Services.

† Female, *n* = 218;

‡ Male, *n* = 50;

§ Total, *n* = 268.

In a subset of 100 samples, the highest agreement was observed between PIMA™ analysers and FACS Calibur as evidenced by smaller mean bias of 7.52 and narrower BA limits of agreement from -111 to 126 and a correlation of 0.97 (Table 2). Wider BA limits of agreement (from -216 to 176 mean bias -20.3) were observed between the FACS Calibur versus NHLS with a correlation of 0.92 compared to PIMA™ analysers versus NHLS (BA limits of agreement from -226 to 200 mean bias -12.78) with a correlation of 0.90.

**TABLE 2:** Bland–Altman comparison of PIMA™ analysers versus National Health Laboratory Services versus FACS Calibur.

Measure of agreement	PIMA analysers – NHLS†	PIMA analysers – FACS Calibur†	FACS Calibur – NHLS†
Mean bias (± 1 s.d.)	-12.78 ± 106.63	7.52 ± 59.26	-20.3 ± 97.97
95% CI bias	-33.94–8.38	-4.24–19.28	-39.74 – -0.86
BA 95% LOA	-226.04–200.48	-111.01–126.05	-216.23–175.63
% Similarity to predicate (%SIM Mean ± s.d.)	101.3 ± 15	103.1 ± 12.7	98.7 ± 11.9

NHLS, National Health Laboratory Services; BA, Bland-Altman; LOA, limits of agreement.

† *n* = 100.

**FIGURE 1:**
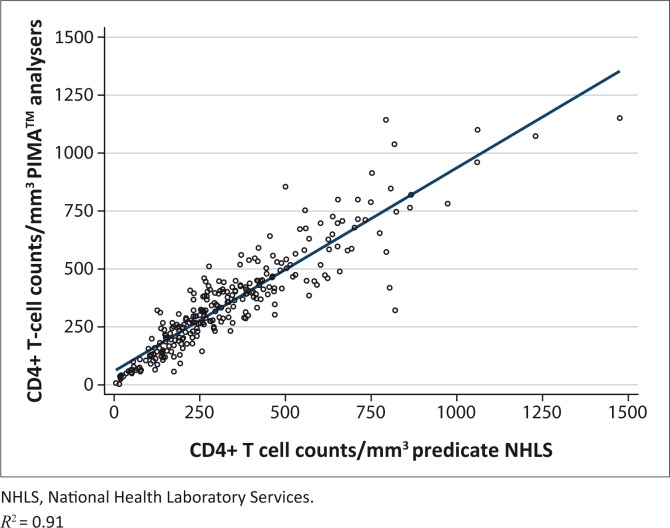
Comparison of CD4+ T-cell counts obtained by the PIMA™ analysers and the National Health Laboratory Services in whole-blood samples.

**FIGURE 2:**
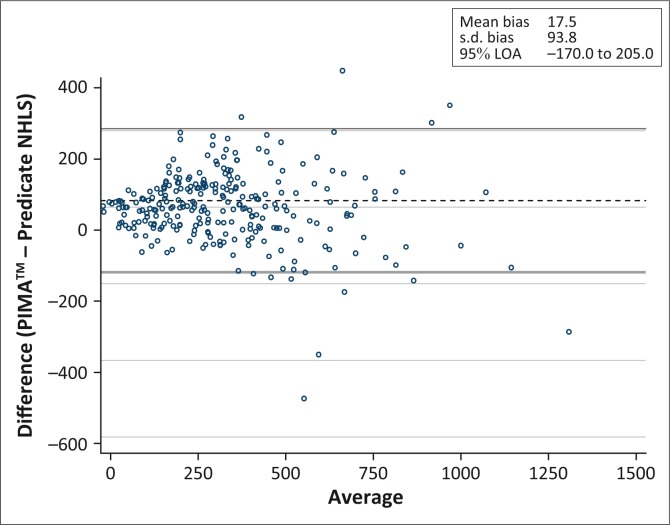
Bland–Altman plot PIMA™ point-of-care analyser – National Health Laboratory Services versus the average of PIMA™ point-of-care analyser and National Health Laboratory Services.

An overall correlation of 0.91 in CD4+ T-cell counts between the PIMA™ analysers and NHLS was observed (Figure 1). The overall mean difference of PIMA™ analysers NHLS was 17.5 cells/mm^3^ (95% confidence interval [CI] 6.2–28.8) (Table 3; Figure 2). When stratified by the following CD4+ T-cell counts: ≤ 350 cells/mm^3^, 351 cells/mm^3^ – 500 cells/mm^3^, ≤ 500 cells/mm^3^ and > 500 cells/mm^3^, the mean difference of PIMA™ analysers – NHLS was 33 cells/mm^3^ (95% CI 23–42), 22 cells/mm^3^ (95% CI -3.5–47), 30 cells/mm^3^ (95% CI 21–39) and -36 cells/mm^3^ (95% CI -78– 6.1), respectively. Acceptable mean percentage similarity in the range of 95% – 110%, with %SIM CVs < 15%, was observed at all CD4+ T-cell count ranges.

**TABLE 3:** Comparison of PIMA™ analysers versus National Health Laboratory Services as categorised by CD4+ T-cell counts.

Measure of agreement	≤ 350 cells/mm^3^†	351 cells/mm – 500 cells/mm^3^‡	≤ 500 cells/mm^3^§	> 500 cells/mm^3^¶	All CD4+ T-cell counts††
Median bias	21	13	21	-23	18
BA bias (± 1 s.d.)	32.9 ± 61.0	21.5 ± 90.8	30.1 ± 69.4	-36.1±150.04	17.5 ±93.8
95% CI bias	23.4–42.3	-3.5–46.6	20.8–39.4	-78.3–6.1	6.2–28.8
BA 95% LOA	-89.2 –154.9	-160.0–203.0	-108.7–168.9	-336.2–263.9	-170.0–205.0
% Similarity to predicate (% SIM mean ± s.d.)	107.4 ± 15.2	102.7 ± 10.5	106.2 ± 14.35	97.97 ± 10	106 ± 15.5
%SIM CV	14.20%	10.20%	13.50%	10.20%	14.60%

BA, Bland-Altman; LOA, limits of agreement.

† *n* = 164;

‡ *n* = 53;

§ *n* = 217;

¶ *n* = 51;

†† *n* = 268.

Under previous SA ART guidelines of ≤ 200 cells/mm^3^ and ≤ 350 cells/mm^3^, the PIMA™ POC analysers displayed a sensitivity and specificity of 73.5%/98.4% and 83.5%/92.3%, respectively (Table 4). Under the current SA guidelines of ≤ 500 CD4+ T-cells/mm^3^, a high sensitivity of 94% and PPV of 95% was observed at the sacrifice of lower specificity of 78%. In the 13 FNs with ≤ 500 cells/mm^3^, the mean bias was 149 CD4+ T-cells/mm^3^.

**TABLE 4:** Performance of PIMA™ analysers compared to National Health Laboratory Services at different CD4+ T-cell thresholds.

CD4+ T-cells/mm^3^	Sensitivity (%)	Specificity (%)	Number misclassified	Number misclassified (%)	Correctly classified (%)	FP Rate	FN Rate	Negative predictive value (%)	Positive predictive value (%)
≤ 200	73.50	98.40	25	9.3	90.7	3/25	22/25	85.20	95.30
≤ 350	83.50	92.30	35	13.0	87.0	8/35	27/35	78.10	94.50
≤ 500	94.00	78.40	24	9.0	91.0	11/24	13/24	75.50	94.90

Note: PIMA™ point-of-care [POC] testing for CD4 counts in predicting antiretroviral initiation in HIV-infected individuals in KwaZulu-Natal, Durban, South Africa.

FP, false-positive; FN, false-negative.

**FIGURE 3:**
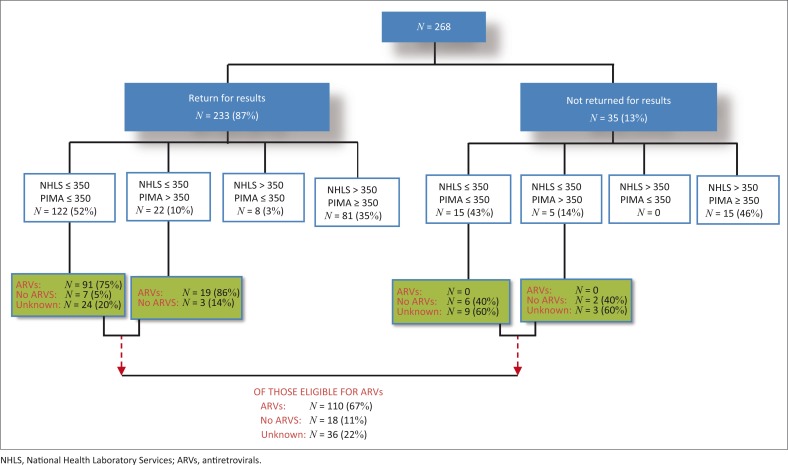
Comparison of CD4+ T-cell counts with respect to antiretroviral therapy eligibility by PIMA™ point-of-care analyser versus National Health Laboratory Services in those HIV-1-infected patients who returned and did not return for their results.

As the study was conducted during 2013, linkage to care data is presented according to the NHLS laboratory CD4 test result of ≤ 350 cells/mm^3^,^25^ 164/268 (61%) of patients were eligible for ART on the day of HCT compared to 145/268 (54%) with the PIMA™ analyser POC CD4 test (Figure 3). The majority of patients (87%) returned to the Lancers Road PHC for their CD4 test result. However, according to the ART register at Lancers Road PHC, 110/164 (67%) of eligible patients were initiated on ART. Of the 35 individuals who did not return to the clinic for their CD4 test result, 20 were eligible (according to the NHLS CD4 result), and not initiated on ART. The median time taken for patients to return for CD4 results was 8 days (IQR 7–14 days) and 7 days (IQR 7–11 days) in those with ≤ 200 cells/mm^3^.

The median time to ART initiation from date of CD4 test was: 49 days (IQR 42–64 regardless of CD4+ T-cell count; 36–63 days in those with ≤ 200 cells/mm^3^).

## Discussion

Conventional flow cytometry to determine CD4 counts usually requires that samples be sent to a central laboratory, which may be off-site. Although the turn-around time for a CD4 test result by the NHLS is 24–72 hours, HIV-1-infected patients are counselled to return to the PHC within 1 week for receipt of these results. POC technologies can reduce these delays resulting in rapid linkage to care. This study demonstrated a high PPV and sensitivity and acceptable specificity in predicting ART eligibility (≤ 500 cells/mm^3^) using the PIMA™ POC analyser as compared to the NHLS CD4 test.

The majority of HIV-1-positive individuals undergoing CD4 testing were women, of whom 25% were 25–29 years old, whereas the majority of men were older than 30 years of age. There were no significant differences in the median CD4+ T-cell count in men versus women performed by the NHLS versus the PIMA™ POC analyser, although the median count was higher in the latter. Overall, according to NHLS versus PIMA™ POC, 81% versus 80% of individuals were eligible for ART initiation (≤ 500 cells/mm^3^), of whom 82% versus 84% were men and 81% versus 79% were women, respectively.

There was high reproducibility in all three PIMA™ POC analysers using normal and low beads with coefficient of variation < 5% over time (10 and 165 days). The PIMA™ POC analyser slightly overestimates NHLS flow cytometry in CD4+ T-cell enumeration in this study, which corroborates most studies using capillary or venous blood.^26,30,31,32^ This overestimation is minimal (mean bias 17 cells/mm^3^) and is not clinically significant. Differences have been reported on conventional CD4 testing platforms between the BD FACS count versus the BD FACS Calibur^23^ where the mean bias between the two platforms was -76 cells/mm^3^ (95% CI LOA -316.0–163.0).

The adequate correlation between the PIMA™ POC analyser and FACS Calibur (0.97) corroborates similar findings in another study.^21^ Although a correlation of > 0.90 was observed between the three platforms, these differences are due to variability of instrument settings, antibodies and fluorochromes used, gating strategies and sample volume input.

The overall sensitivity of the PIMA™ POC CD4 test in HIV-1-infected adults and pregnant mothers to determine their eligibility for ART has been reported at 96.3% in individuals with a CD4+ T-cell count of ≤ 250 cells/mm^3^,^24^ and 92% and 91% in those with ≤ 350 cells/mm^3^,^20,22^. The total misclassifications have been documented in several studies using the PIMA™ POC analyser: 31%,^18^ 17%,^12^ 5.2%,^33^ 6.7% – 14%,^31^ 10%,^22^ 11.4%^34^ and 9%.^19^ This study found 13% misclassifications, of which 27/35 were FNs at ≤ 350 CD4+ T-cells/mm^3^. At a CD4+ T-cell threshold of ≤ 500 cells/mm^3^, 91% of patients were correctly classified as either eligible or ineligible for ART. In the 13 FNs, the mean bias observed was 149 cells/mm^3^. The PPV of 95% indicates that only 5% of those who are diagnosed as eligible for ART according to the PIMA™ POC analyser would not be needing treatment according to the NHLS CD4 test result. A high sensitivity of 94% was observed at the sacrifice of lower specificity of 78%. This high sensitivity corroborates recent findings^21,22,34^ and fits in well with current SA HIV and AIDS guidelines^35^ where all those eligible for treatment will be initiated but it will come at a cost of low specificity, whereby individuals not needing treatment will be commenced on ART. However, in light of ART-lowering viral loads and reducing horizontal transmission,^36,37^ this downside is minimised. A recent study has demonstrated that as household ART coverage is increased, there is a decrease in HIV acquisition.^38^ The agreement in these data between the PIMA™ POC analyser and NHLS laboratory-based flow cytometry appears to decline with increasing CD4+ T-cell count ≥ 500 cells/mm^3^. This is not of concern as these HIV-1-infected individuals are ineligible for ART under current guidelines.

From the operational perspective in the use of the PIMA™ POC analyser, similar to other studies using venous or capillary blood,^18,20,21,22,33^ we also experienced reading errors (8%) mostly because of movement and vibration.^34^ The ‘operator’ used in our study was a trained laboratory technician compared to health professionals, for example, nurse or counsellor. Several studies have reported that the PIMA™ POC is interchangeable with conventional platforms,^18,20,22,30,31,33,39^ although a study in Kenya^23^ found it to be unreliable due to the high coefficient of repeatability and misclassification in favour of undertreatment compared to the FACS Calibur.

Under the standard SA HIV and AIDS guidelines operating at the time of the study, we observed that the median time for patients to return for their CD4 results was 8 days and 7 days in those with ≤ 200 cells/mm^3^, with a median of 49 days regardless of CD4+ T-cell count from CD4 testing to ART initiation. The use of the PIMA™ POC analysers could facilitate the fast tracking of patients with CD4+ T-cell count ≤ 200 cells/mm^3^ onto ART within 7 days. In this study, the provision of immediate CD4 test results to patients would have prevented the 35/268 not having access to their results (through them not returning), in whom over half (57%) were eligible for ART.

The high rates (61%) of ‘walk–in’ patients found in this study who were eligible (≤ 350 cells/mm^3^) for immediate ART at the time of the HIV test, half of whom had CD4+ T-cell counts ≤ 248 cells/mm^3^, and the time lapse to ART initiation undergirds the urgent need for the use of the rapid PIMA™ POC technology. At a threshold of ≤ 500 cell/mm^3^, 75% of patients had a median CD4+ T-cell count of 444 cells/mm^3^ at the time of the HIV test. A recent study reported that providing same-day POC CD4 testing that is not rapid has no benefit in health outcomes.^9^ As suggested by others,^11,18,40,41^ we agree that using existing infrastructure and based on demand, the integration of a PHC POC mini-laboratory run by dedicated personnel (laboratory technician) is possible, offering tests for staging and pathology that assess ART eligibility.^42,43^ However, as suggested in a recent systematic review,^44^ this needs to be supported by streamlining services through minimising patient clinic visits,^22^ addressing psychosocial issues and barriers to healthcare,^45^ optimising the opportunity for patient empowerment through counselling and peer support,^46^ emphasising the importance of starting and adhering to ART if eligible,^6^ positive health-seeking behaviours and encouragement for patient ownership of their health. A family-centred model of integrated healthcare incorporating most of the above-mentioned health system changes has previously been shown, in a similar population, to yield high adherence (94%) and retention in the care and management of HIV-1-positive individuals.^47,48^ In this study, similar reasons for not linking into care were given as found previously^45^; of those eligible for ART who did not access treatment (33%), the reasons given upon telephonic communication were economic (no money to cover transport costs), social (too busy to come to the clinic), structural (cannot take time off work) and emotional (were not ready to take ART and they were still feeling well). We would anticipate that there would be an increase in loss of uptake of care at the higher CD4+ T-cell count threshold of ≤ 500 cells/mm^3^ because of the reasons re-iterated. Previous studies have shown that provision of immediate CD4+ T-cell count results increased the number of patients linking into care.^2,4,6,11,13^

## Conclusion

In summary, the overall agreement between PIMA™ POC analyser and NHLS CD4+ T-cell count enumeration in adult HIV-1-positive individuals was acceptable with clinically insignificant mean bias. Together with high PPV and sensitivity and acceptable specificity, the PIMA™ POC CD4 test has the potential role for CD4+ T-cell enumeration in PHC settings and lends itself to be an excellent facilitator in rapid linkage to care in ART programmes, particularly that it has been demonstrated in simulated cohort models of HIV-1-infected adults and pregnant women, to result in not only better clinical outcomes but also to cost savings in the long term.^49,50^ Even in the era of ‘test and treat’,^51^ PIMA™ POC CD4 testing would facilitate the fast tracking of patients with low CD4+ T-cell counts (< 200 cells/mm^3^) for the administration of cotrimoxazole prophylaxis as well as in screening for cryptococcal infection in patients with < 100 cells/mm^3^. The operational role of the PIMA™ POC CD4 test in provision of immediate CD4+ T-cell count results combined with integrated health system changes and interventions such as mobile phone technology and provision of incentives need to be evaluated in a variety of settings across the HIV cascade, to determine its implementation effectiveness in linkage to care, time to ART initiation and retention in HIV care.
